# Global agricultural concept space: lightweight semantics for pragmatic interoperability

**DOI:** 10.1038/s41538-019-0048-6

**Published:** 2019-09-18

**Authors:** Thomas Baker, Brandon Whitehead, Ruthie Musker, Johannes Keizer

**Affiliations:** 1 Plain Semantics, Bonn, Germany; 2 CAB International, Wallingford, UK; 3 Global Open Data for Agriculture and Nutrition (GODAN) Secretariat, Wallingford, UK

**Keywords:** Agriculture, Developing world

## Abstract

Progress on research and innovation in food technology depends increasingly on the use of structured vocabularies—concept schemes, thesauri, and ontologies—for discovering and re-using a diversity of data sources. Here, we report on GACS Core, a concept scheme in the larger Global Agricultural Concept Space (GACS), which was formed by mapping between the most frequently used concepts of AGROVOC, CAB Thesaurus, and NAL Thesaurus and serves as a target for mapping near-equivalent concepts from other vocabularies. It provides globally unique identifiers, which can be used as keywords in bibliographic databases, tags for web content, for building lightweight facet schemes, and for annotating spreadsheets, databases, and image metadata using synonyms and variant labels in 25 languages. The minimal semantics of GACS allows terms defined with more precision in ontologies, or less precision in controlled vocabularies, to be linked together making it easier to discover and integrate semantically diverse data sources.

## Introduction

Sustainable agricultural value chains and global food security cannot be achieved without intelligent use and re-use of data. Data impact increases by an order of magnitude when the information is mapped to a common descriptive framework—semantics—in which both humans and machines make use of data by leveraging relationships within, and between, datasets. These relationships allow for faster and effective decision-making while increasing the reproducibility, transfer and impact of scientific discoveries.^1^

Research and innovation in food technology depend increasingly on “Semantic Web” vocabularies—sets of terms identified with globally unique Web addresses (Uniform Resource Identifiers, or URIs) and made available on the open Web. URIs provide language-neutral, globally valid names for concepts, which can be used in a variety of applications and in all phases of research and discovery.

This paper describes Global Agricultural Concept Space (GACS), a namespace of concepts relevant to food and agriculture, and the choices made in designing its first concept scheme, GACS Core. GACS Core was created as a mapping target for the concepts most frequently used in three current, long-standing, concept schemes: AGROVOC (http://aims.fao.org/agrovoc), CAB Thesaurus (https://www.cabi.org/cabthesaurus), and National Agricultural Library (NAL) Thesaurus (https://agclass.nal.usda.gov). These three concept schemes are used by their respective institutions to index over 25 million bibliographic records, as well as myriad institutions and agencies in their applications.

GACS Core provides globally unique identifiers, with synonyms and variant labels in up to 25 languages, usable as tags or keywords for indexing text resources, building lightweight facet schemes, and annotating spreadsheets, databases, and image metadata, to enable broad-brush discovery. Its concepts serve as mapping targets for equivalent and near-equivalent concepts in related knowledge organization systems, text labels in controlled vocabularies, formal ontologies, or other concept schemes as a basis for annotating and discovering data.

GACS Core is modeled using the Simple Knowledge Organization System (SKOS), a knowledge representation language that was designed for expressing deliberately lightweight semantics. SKOS concepts schemes provide pragmatic interoperability by accommodating semantic diversity and tolerating near-equivalences in support of broad-brush resource discovery. Discovery is not limited to traditional research artifacts like bibliographic databases, but includes, for example, spreadsheets of agricultural field data, crop image databases, other lightweight semantic resources (e.g., term lists, controlled vocabularies, etc.) or even concepts defined with more precision in domain-specific ontologies.^2^

**Fig. 1:**
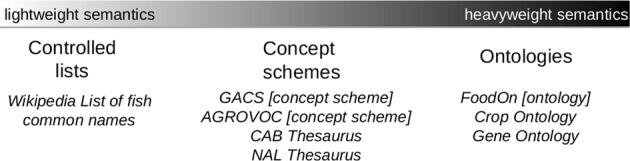
Semantic spectrum with Agri-Food examples. The spectrum illustrates lighter semantics on the left with increasingly more precision and complexity, in the form of shared understanding and logic, as one moves to the right

A concept scheme is contrasted here to the design of semantically more complex domain ontologies (see Fig. 1 and Table 1). Domain ontologies are designed to support intelligent applications to make decisions,^3^ suggest diagnoses,^4^ or answer complex queries.^5^ Such logical operations are based on a selective caricature of reality—“an abstract, simplified view of the world”^6^—encoded in a vocabulary of properties and classes expressed using a formal logic, which allows a machine to derive inferences from the axioms. For example, FoodOn, a semantically more precise ontology, models the class of mammary glands as a mathematical subset of a super-class, “animal body or body part.”

**Table 1:** The SKOS to OWL continuum

	Concept schemes		Ontologies (OWL)
	SKOS	SKOS with extensions	
When you want to	Semantically enable a knowledge organization system. Query on data patterns.	Extend SKOS with custom relations, concept types, or facet hierarchies.	Automate decisions. Query by inferencing on a precise domain model.
In order to	Annotate “non-semantic” data for discovery across languages. Annotate ontologies.	Enable more complex navigation, consistency checks, and queries.	Annotate “non-semantic” data with precise types or qualities.
For capturing	A general consensus within or across communities of practice.		Expert consensus on a specific view of reality.
Maintenance cost	Low-to-medium		High
Examples discussed in this paper	Simple GACS concept schemes (future)	GACS Core, AGROVOC, NALT, CABT	FoodOn, Crop Ontology

GACS Core, in contrast, defines a chain of generically broader concepts relating mammary glands to animal organs without specifying how the concepts relate in terms of mathematical set theory. This relative lack of precise semantics minimizes the maintenance costs of GACS Core and maximizes its potential for re-use across a broad range of applications.

GACS is the first step to creating a space for interconnected, interoperable, semantic assets relevant to agriculture and food security. GACS affords an interoperable layer transforming massive data silos to a more reusable web of data and services by making previously hidden or obscure resources more easily discoverable.

## Results

The design of GACS Core was guided by three requirements:

- *Persistent.* Once coined, the URI of a concept can be moved in or out of a specific concept scheme or assigned a status of deprecated, never simply deleted, and its meaning remains fundamentally stable. Pragmatically, it means that older or less frequently updated services will continue to function as expected even if concepts are flagged as deprecated.
- *Re-usable.* GACS Core was designed for pragmatic interoperability across a diversity of fields and multiple languages, with minimal relationships and labels sufficient for supporting disambiguation and simple consistency checks. GACS Core also facilitates re-usability of other data and resources.
- *Minimally maintainable.* GACS Core was designed to be maintainable with minimal effort. Its set of terms was selected primarily on the basis of their frequency of use in databases indexed by the three source thesauri, and its semantic structure was limited to constructs that would be easy for future maintainers to understand and to apply with consistency.

### The semantics of GACS Core

**Fig. 2:**
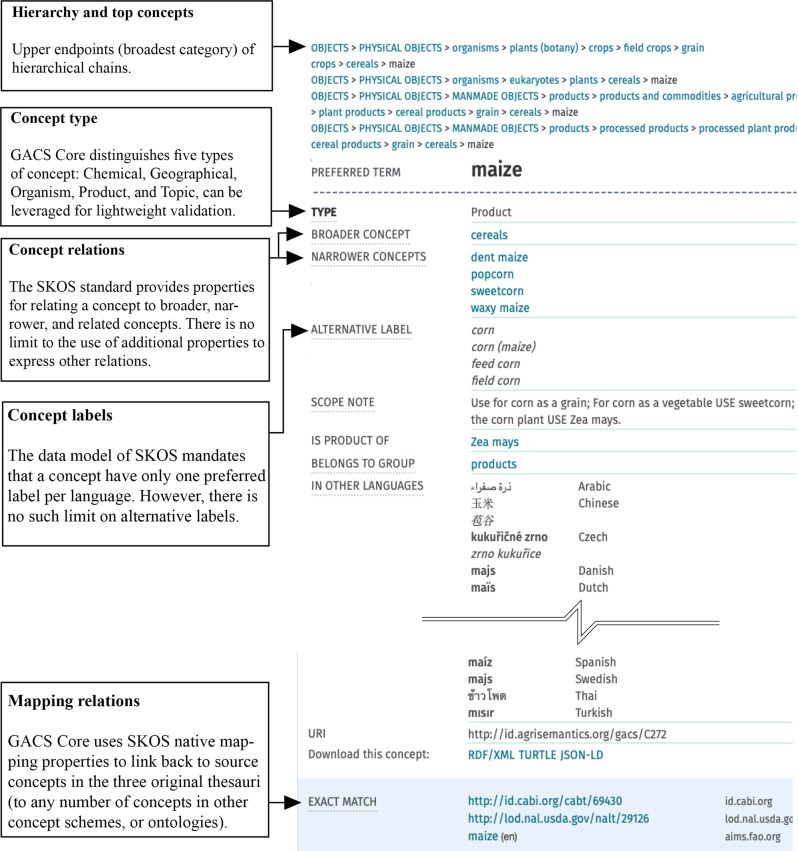
The concept “Maize”, as rendered by Skosmos in a browser, in main figure on right (see: http://browser.agrisemantics.org/gacs/en/page/C272). The left sidebar describes what is being rendered from the SKOS encoding of GACS Core

**Fig. 3:**
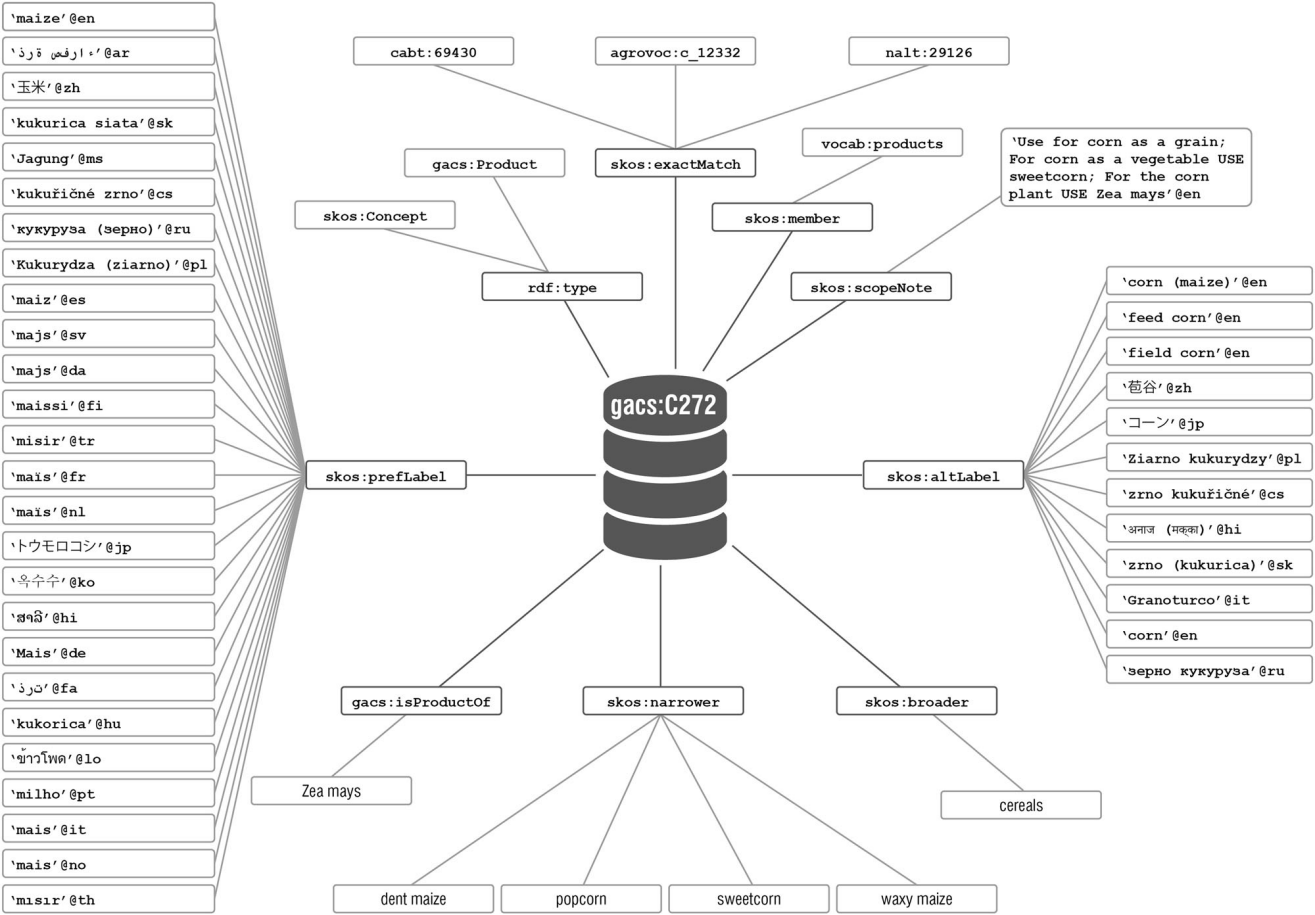
GACS schema represented graphically using “maize” as an example

GACS Core is defined by lightweight semantics in accordance with the SKOS data model.^7,8^ Concepts are defined not just by natural-language labels and definitions, if available, but by the semantic contexts in which they are embedded. This context consists of (see Figs 2 and 3):

- *Hierarchy and top concepts.* In thesaurus practice, top concepts typically serve as the upper endpoints, or broadest category, of hierarchical chains, ideally of transitive “is a” relations (as in: dog is a mammal, mammal is an animal, therefore dog is an animal). GACS Core has three top concepts adapted from the Finnish General Upper Ontology^9^ (YSO): *Objects*, *Events and Actions*, *and Properties*–concepts intuitively understandable, at a first approximation, as nouns, verbs, and adjectives.
- *Thematic groups.* Thematic groups provide a quick way for a user to grasp its scope. The GACS team adapted the CAB Classified Thesaurus, a product of prior cooperation among Food and Agricultural Organization (FAO), CAB International (CABI), and NAL in the 1990s, for grouping concepts under scientific fields such as *Physical Sciences*, *Earth Sciences*, and *Life Sciences*.^10^
- *Concept relations.* The SKOS standard provides properties for relating a concept to broader, narrower, and related concepts but there is no limit to the use of additional properties to express other relations. The GACS team opted to create just one pair of additional (custom) relation properties: gacs:hasProduct and gacs:productOf to relate, for example, *maize* as a grain cereal (product) to *Zea mays* as a eukaryotic plant (organism).
- *Concept types.* GACS Core distinguishes five types of concept: *Chemical*, *Geographical*, *Organism*, *Product*, and *Topic*—a minimal set of generic types for exploring the benefits of concept typing before committing to anything more granular. Concept types can be leveraged for validation, for example to verify that gacs:hasProduct and gacs:productOf are being used correctly. The concept types, expressed as sub-classes of skos:Concept, can be used to pull together concepts from across the hierarchy.
- *Scientific and common names*. Scientific names are flagged in AGROVOC and CAB Thesaurus by distinguishing types of label. Instead of taking on more complex extensions (i.e., SKOS XL^8^), the GACS team opted to simply flag scientific names with their own unique language tag, @zxx-x-taxon. Similar to other language tags, @en, @fr, etc., the unique language tag allows users to retrieve and use scientific names, specifically, if needed – i.e., “Zea Mays”@zxx-x-taxon is the scientific name for *“*corn (plant)”.
- *Concept labels*. The data model of SKOS, like the thesaurus standards on which it is based, mandates that a concept have only one preferred label per language. However, there is no such limit on alternative labels, which can richly annotate a concept with variant spellings, regional designations, and the like. Multiple labels improve findability by situating each concept in its own multilingual word cloud.
- *Mapping relations*. GACS Core uses SKOS native mapping properties to link back to source concepts in the three original thesauri and potentially to any number of concepts in other concept schemes, controlled vocabularies, and ontologies.

### Building the Global Agricultural Concept Space

GACS Core is but the first of many potential concept schemes to be defined and maintained in the GACS. It will be maintained as a set of high-level, generic, frequently used concepts, with high guarantees of quality and semantic stability implicit in the cross-mapping of the three source thesauri. Its governance model involves the three organizations that collaborated in its creation (FAO, NAL, and CABI), and CABI has committed to working with partners to periodically verify the validity of mappings to AGROVOC, NAL Thesaurus, and CAB Thesaurus, respectively.

GACS concepts are also intended to serve as building blocks, freely available to any interested organization or user, for the construction of concept schemes, lists, classifications, or ontologies outside of GACS. GACS provides a namespace for concept schemes on specific topics, such as crops, which may be curated by separate editorial boards. The policies governing this process encourage the sharing of concepts between overlapping concept schemes, where appropriate, and the creation of mappings to narrower and broader concepts in the concept space.

The current iteration of GACS has been released under a Creative Commons license (https://creativecommons.org/licenses/by/4.0) and is available both as a static download (https://agrisemantics.org/GACS) and via a version control repository (https://github.com/gacs/gacs-scheme)—to ease technical integration and update notifications for applications. GACS is registered in the Linked Open Data (LOD) Cloud (https://www.lod-cloud.net) and an openly accessible SPARQL endpoint, for real-time programmatic access, is in development.

## Discussion

Ontologies became popular with the publication of the Web Ontology Language^11^ (OWL) as a W3C Recommendation in 2004. At that time, ontologies appeared to offer a path for porting traditional knowledge organization systems—the classification systems and terminological thesauri that had been developed by many institutions, sometimes over many decades, to organize their data—to the Semantic Web.

Also in 2004, the maintainers of AGROVOC (or “AIMS team”) began the task of re-engineering AGROVOC from a thesaurus to a “fully fledged ontology”. A more precisely specified ontology, it was hoped, would support more intelligent queries: for example, to determine whether a specific farming method had been used in a dryland area for a given crop. To this end, 179 custom relation properties were coined, such as agrovoc:hasComponent for relating an animal to a body part and agrovoc:hasSpellingVariant for relating one label to another.^12^

However, a study of AGROVOC users 6 years later found little support for the use of these custom relation properties.^13^ In the absence of specific tools and requirements for reasoning, it was unclear to some users what purpose they served. One respondent told of colleagues who tried to make an application to help farmers diagnose plant diseases. Despite their sophisticated understanding of plants and pesticides, they were unable to use this knowledge to build an *intelligent* system. In the end, for the 32,000 concepts of AGROVOC, eleven concept relations and eleven label relations are used more than 500 times, and two-thirds form a long tail of properties used less than twenty times.^14^

The AIMS team also drew lessons from its participation in NeOn (2006–2010), a multinational European project about using ontologies for large-scale applications in distributed environments, where they helped implement a prototype decision support system in support of the long-term goal of sustainable fisheries. The task required integrating data about fishing areas, fish species, commodities, vessels, and fishing gear, with images, into a queryable whole.

The process of aligning a network of independently evolving ontologies proved to be time-consuming and error-prone. Alignments were especially problematic where ontologies were based on different models. When fish species were modeled as classes, with actual fish as instances, species needed to be pragmatically converted to instances for the purposes of mapping to statistical time series. Distinguishing classes from instances in a logically sound way, a project report concluded, “would require a huge amount of fishery experts time, and only after they are organized in a team sided by ontology designers and are taught design tools adequately”.^15^

The value of an ontology lies in the precision with which it encodes a specific interpretation of reality. FoodOn, for example, aims at representing knowledge about food and food processes comprehensively enough to drive applications in areas such as food safety, farm-to-fork traceability, and intelligent kitchens.^3,16^ FoodOn encodes expert consensus about complex interrelations within food systems so that machines can compute logical inferences, for example to categorize foods based on their properties. Questions cannot automatically be answered, nor objects classified, diagnoses provided, or decisions taken, reliably, unless the ontology presents a well-defined point of view designed and engineered for specific goals.

However, what makes ontologies such as FoodOn so powerful for logic-based computation is precisely what makes them so expensive to create and maintain. Its classes are the object of an ongoing process of axiomatization, where candidate axioms must carefully be fitted into a mathematically logical hierarchy of related classes. The knowledge encoded in such ontologies must continually be reviewed and revised by experts. This can be problematic where communities of experts differ on what to describe, with what model, or even on the facts themselves. As a concept scheme, limited by design to a handful of logical distinctions, GACS is better-suited for broad-brush resource discovery, and its relative simplicity makes it less expensive to create and maintain.

The design of SKOS, published as a W3C Recommendation in 2009, specifically addresses the risk of incorrect use by avoiding the sort of semantic baggage that can create false precision or unintended logical contradictions in heavyweight ontologies. It was guided by the principle of “minimal semantic commitment”, whereby it limits its assertions to the minimum required by its intended uses—the “weakest theory”—leaving it to users to specialize its vocabulary as needed.^6^ The hierarchies and association networks of a SKOS concept scheme were not intended to be reliably interpreted as formal axioms or facts about the world.^8^

SKOS has solved some of the issues raised by inappropriate uses of OWL, such as false ontological precision, and provided a basis for pragmatic interoperability. Like a thesaurus, a SKOS concept scheme is optimized for organizing and finding relevant objects, such as documents, in a given domain.^17,18^

SKOS concept schemes can be generated from OWL ontologies automatically, incurring little cost beyond that of maintaining the source ontologies. An informally defined KOS, however, cannot be converted automatically into OWL, with its formal semantics, without risking the introduction of false precision.^19^ Hierarchical relationships, for example, may need to be disambiguated into relationships of class instantiation, class subsumption, or of parts and wholes. Tools alone cannot impart principles of good design or prevent modelers from casually combining terms from multiple ontologies, based on different models of the world, into inconsistent “Frankenstein ontologies”.^20^

The uptake of SKOS prior to its finalization as a W3C Recommendation coincided with a shift in discourse, starting in 2006, away from Semantic Web towards the more accessible goal of Linked Data.^21^ Starting with a cloud of data sources clustered around a database extracted from Wikipedia, a Linked Data movement grew by taking a more inclusive view of data technologies and recasting Resource Description Framework (RDF) as a language for facilitating interoperability among data sources. The Linked Data vision valued pragmatic re-usability over formalized semantics, tolerated ambiguity in place of semantic precision, and accepted partial interoperability as the only goal that is realistically attainable in a massively diverse web of data.

In agricultural research, the re-use of datasets is limited by the sheer effort required to determine equivalences among differently named elements embedded in a broad diversity of applications. However, when used to annotate datasets, the GACS Core URI http://id.agrisemantics.org/gacs/C9983 can relate spreadsheet values in Lab A, “Zebrafish” and “diazinon”, to equivalent database values in Lab B, “Danio rerio” and “二嗪磷“ (“diazinon” in Chinese), and again to metadata tags in an image repository in Lab C, “Brachydanio rerio” and “ديازينون” (“diazinon” in Arabic), providing a queryable link, in the form of a web URI, as a semantic entry point to previously non-semantic data elements.

By providing pragmatic links to other concept schemes, to the literature, to ontologies, and to datasets, the semantically weak but richly linked concepts of the GACS can improve the coherence of agricultural research and contribute to the ultimate goal of ensuring our food security.

**Fig. 4:**
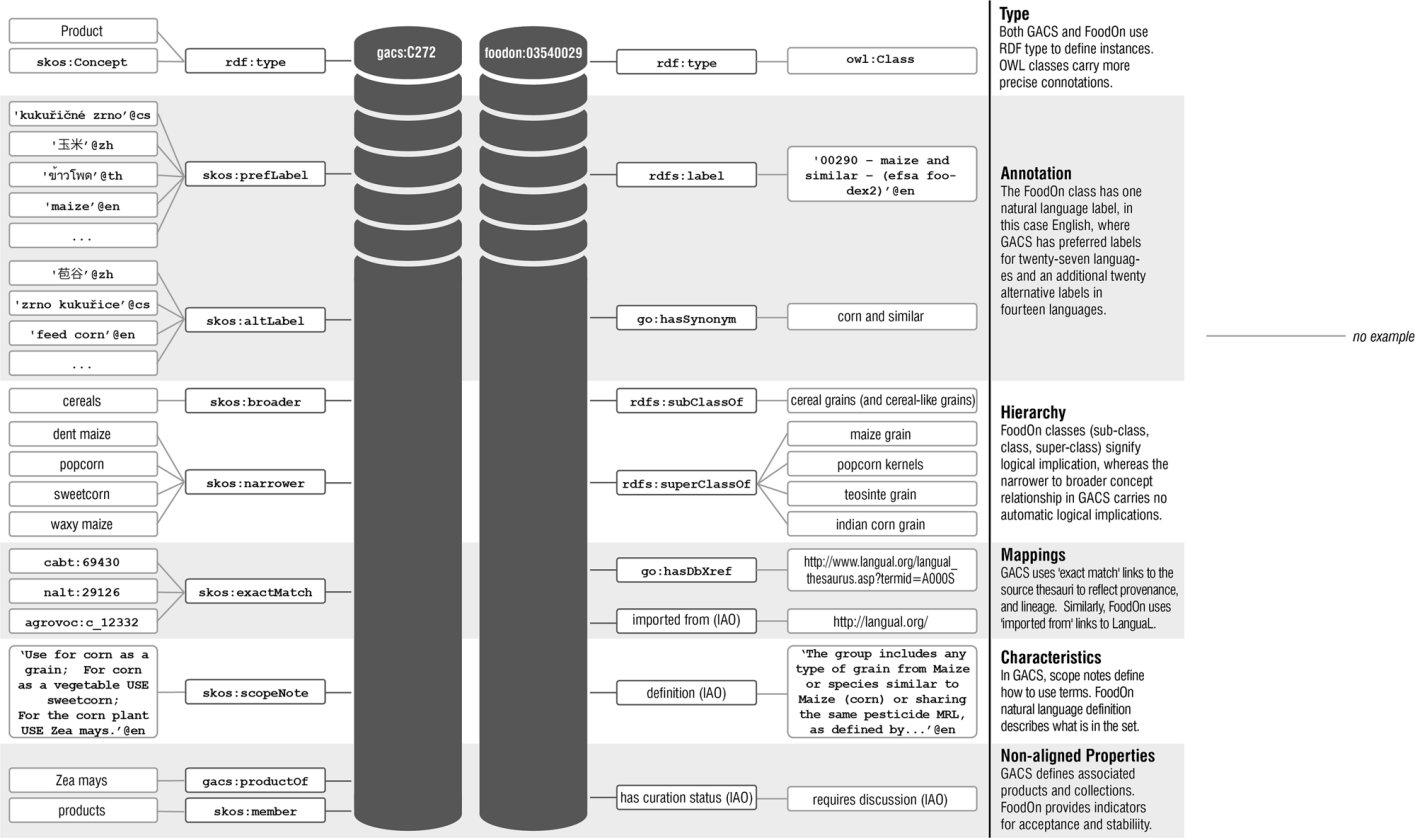
A side by side visualization of GACS and FoodOn data (properties and values) using comparable maize concepts. Labels are shown in quotes with language tags; classes are shown in natural-language without quotes

GACS and FoodOn are intended for different purposes. As shown on the example of “maize” in Fig. 4, GACS depicts a domain of discourse: its concepts, relationships among those concepts (including the relationship between product and organism), thematic groupings of concepts, and the multitude of natural-language terms with which the concepts are labeled. FoodOn, which is scoped more specifically to aspects of maize that are relevant to the traceability of food in the supply chain, focuses on relationships between the grain itself, derived food products, related crops and production processes. With their complementary roles, both serve the greater purpose of supporting the improvement of agriculture and food security.

## Methods

After the Food and Agricultural Organization of the United Nations (FAO), CAB International (CABI), and the USDA National Agricultural Library (NAL) agreed to collaborate in 2013, the process of creating a Global Agricultural Concept Scheme (the original meaning of GACS) begin in March 2014 with the formation of a joint working group consisting of the thesaurus managers for AGROVOC, NAL Thesaurus, and CAB Thesaurus, with the help of two consultants.

A feasibility study found that some 98% of the indexing fields in AGRIS used just 10,000 out of the 32,000-plus concepts in AGROVOC, so mapping began with sets of the 10,000 most frequently used concepts from each thesaurus. These sets were algorithmically mapped to each other, pairwise, using the AgreementMakerLight system for matching ontologies.^22^ The mappings were loaded into Google spreadsheets and manually verified. The verified mappings were scanned for clusters of inconsistent mappings.^23^ The clusters were discussed and resolved in face-to-face meetings and teleconferences. The corrected mappings were used to generate new concepts for GACS. This iteratively generated concept scheme was deemed ready for a soft launch in May 2016 for use by early adopters with 15,000 concepts, labeled with 350,000 terms in more than twenty-five languages, under the name GACS Core Beta 3.1.^24,25^

Each new concept created for GACS inherited hierarchical contexts from up to three source concepts, so almost one third of the concepts in GACS ended up with more than one broader concept (polyhierarchy). While a certain measure of polyhierarchy may be inevitable, even desirable, the thesaurus ideal is to keep hierarchies as simple and pyramid-like as possible. The polyhierarchy of GACS Core Beta 3.1 was too expensive to support the formulation of coherent principles that could be sustainably applied going forward.

A workshop sponsored by the Bill and Melinda Gates Foundation in July 2015 re-cast GACS as a hub for clustering concepts of approximately equivalent meaning across a broader landscape of Semantic Web vocabularies and ontologies in agriculture.^26^ In the course of further meetings, the role of GACS as a hub vocabulary was extended to include annotation of the “non-semantic” databases and spreadsheets used for recording agricultural field data.

A survey of 26 GACS stakeholders in November 2016 presented three alternative scenarios for clarifying the GACS hierarchy. The first scenario, with a small number of concepts, was based on YSO. The second, based on AGROVOC, had 25 facet-like top concepts: *Organisms* (by far the most frequent), followed by *Substances* and *Entities*, then by a long tail of lesser-used concepts such as *Events*, *Factors*, *Features*, *Properties*, *Objects*, *Phenomena*, *Strategies*, and *Time*. The third, based on the 1999 CAB Classified Thesaurus, placed concepts under thematic groups.

The survey revealed broad agreement that hierarchy was needed and that all scenarios were in some sense valid, with no clear favorite, but with the caveat that they would all not be equally maintainable. It was decided that the existing hierarchy should be cleaned, leaving enough hierarchy to disambiguate and navigate between concepts, and that the existing thematic groups should be kept as an additional view.

GACS Core was then entrusted to the thesaurus expert Lori Finch of NAL, who systematically checked and corrected the hierarchy, along with thousands of other details, in a Quality Improvement Project from April through November 2017, resulting in a Beta 4.0 release. The 600 top concepts (concepts with no broader concept) were consolidated under just three; broader-narrower relations were checked for typological consistency; and the assignment of concepts to thematic groups was completed. In recognition that shared semantics are key to making open data useful, the GACS Working Group was supported by the initiative for Global Open Data in Agriculture and Nutrition (GODAN).

In 2018, the GACS stakeholders acknowledged this shift in role by redefining the acronym “GACS” to mean Global Agricultural Concept *Space*. Analogously to an RDF namespace—a set of RDF terms identified with common base URI—a “concept space” is a namespace of SKOS concepts.

## Current and future work

Although the initial release is stable, there is planned work to enhance and grow the project. This final section is split between currently planned endeavors and those envisioned in the near future.

The governance of GACS, currently managed by CABI with input from its founding partners, would be well served under a group of stakeholders from a broader community of practice. Topics are centered around processes by which new terms and concepts are added, conflict resolution, and which technologies facilitate a distributed collaboration, while adhering to the main tenets of GACS—persistent, re-usable, and minimally maintainable.

In a time of declining budgets and accelerating scientific change, centralized and generalist maintenance teams struggle to keep pace. At the same time, the ease with which concepts can be mapped over the Web holds out the potential for creating a more efficient division of labor among maintenance communities. The passive maintenance of mappings keeps GACS concepts up-to-date and provides helpful redundancy against the resiliency of external concepts; should they cease to exist or be maintained, the GACS concept from which it is mapped will remain valid.

The GACS team is planning to test the devolution of maintenance responsibility for specific concept types to external authorities. Because of the URI persistence principle by which GACS URIs can never be abandoned, entire categories of URIs will be maintained “passively”, by monitoring changes in concept schemes to which GACS has been mapped and correcting the mappings accordingly. As an example, NAL is exploring ways to reflect a selection of the chemicals cataloged by authoritative domain sources, such as PubChem in the NAL Thesaurus. The GACS team would periodically verify existing mappings to 1500 chemicals and pull in new chemicals from the NAL Thesaurus, as needed, based on frequency of use.

As GACS was originally conceived as a mapping between three source thesauri, other mappings are also welcome, and needed, to achieve a broader scope of interoperability. Existing ontologies such as FoodOn, the Agronomy Ontology (AgrO; https://github.com/AgriculturalSemantics/agro), and the Crop Ontology (http://www.cropontology.org) may find a mapping to GACS concepts allows for increased precision and recall, as well as re-usability, via leveraging the numerous language labels already in the concept space. For example, the concept labeled “maize” in GACS could be related to the FoodOn class labeled “00290—maize and similar—(efsa foodex2)” via a skos:related link. Mappings could potentially be automated, at least to some degree, perhaps expedited by the AgroPortal ontology repository and service (http://agroportal.lirmm.fr). Similarly, employing the conventions discussed by the Biodiversity Information Standards (TDWG) Taxonomic Names and Concepts working group (https://github.com/tdwg/tnc) could begin to reconcile multiple other communities of practice.

Some stakeholders would like to position GACS as the default entry point for semantic search as a multilingual, lexically rich, semantic hub. One such proposal advocates using GACS concepts in the context of an agricultural based extension for Schema.org (https://schema.org). This has been completed for other specific domains (i.e., https://bib.schema.org), but science domains have largely been left to their own accord. Similarly, mapping GACS concepts to Wikidata entities would: (1) allow the community agriculturally contextualized access to a massive open data project, (2) leverage the workforce of the thousands of volunteers involved in that effort, and (3) broaden the set of mappings to everything mapped from Wikidata (https://wikidata.org).

In addition to using GACS as a semantic hub, the D2KAB project (http://d2kab.strikingly.com/) has planned to investigate machine learning approaches using GACS. This will likely involve GACS as a training data set used to classify semantic types within AgroPortal.

The IC^3^-FOODS initiative, which develops authoritative ontologies about food, could improve access to and integration among its ontologies by using GACS concepts as mapping targets for the classes of its ontologies. As discussed at IC-FOODS 2019, for example, IC^3^-FOODS could in principle create and curate a concept scheme for food ingredients within the GACS concept space, re-using existing concepts from GACS Core (e.g., by listing “milk” as an ingredient) and creating new concepts where needed. Such cooperative curation of common semantics would improve the integration and coherence of agricultural initiatives across domains and languages.

### Reporting summary

Further information on research design is available in the Nature Research Reporting Summary linked to this article.

## Supplementary information

reporting summary

## Data Availability

GACS is available both as a static download (https://agrisemantics.org/GACS) and via a version control repository (https://github.com/gacs/gacs-scheme). The three source thesauri, AGROVOC (http://aims.fao.org/agrovoc), CAB Thesaurus (https://www.cabi.org/cabthesaurus), and NAL Thesaurus (https://agclass.nal.usda.gov), are available in various viewers and formats from their respective websites.

## Supplementary information

**Supplementary Information** accompanies the paper on the *npj Science of Food* website (10.1038/s41538-019-0048-6).
